# Pristine PN junction toward atomic layer devices

**DOI:** 10.1038/s41377-022-00814-8

**Published:** 2022-06-06

**Authors:** Hui Xia, Man Luo, Wenjing Wang, Hailu Wang, Tianxin Li, Zhen Wang, Hangyu Xu, Yue Chen, Yong Zhou, Fang Wang, Runzhang Xie, Peng Wang, Weida Hu, Wei Lu

**Affiliations:** 1 State Key Laboratory of Infrared Physics, Shanghai Institute of Technical Physics, Chinese Academy of Sciences, Shanghai, 200083 China; 2 University of Chinese Academy of Sciences, 100049 Beijing, China; 3 Jiangsu Key Laboratory of ASIC Design, School of Information Science and Technology, Nantong University, Nantong, 226019 Jiangsu China; 4 School of Physical Science and Technology, ShanghaiTech University, Shanghai, 201210 China

**Keywords:** Photonic devices, Optical materials and structures

## Abstract

In semiconductor manufacturing, PN junction is formed by introducing dopants to activate neighboring electron and hole conductance. To avoid structural distortion and failure, it generally requires the foreign dopants localize in the designated micro-areas. This, however, is challenging due to an inevitable interdiffusion process. Here we report a brand-new junction architecture, called “layer PN junction”, that might break through such limit and help redefine the semiconductor device architecture. Different from all existing semiconductors, we find that a variety of van der Waals materials are doping themselves from n- to p-type conductance with an increasing/decreasing layer-number. It means the capability of constructing homogeneous PN junctions in monolayers’ dimension/precision, with record high rectification-ratio (>10^5^) and low cut-off current (<1 pA). More importantly, it spawns intriguing functionalities, like gate-switchable-rectification and noise-signal decoupled avalanching. Findings disclosed here might open up a path to develop novel nanodevice applications, where the geometrical size becomes the only critical factor in tuning charge-carrier distribution and thus functionality.

## Introduction

Junctions, including Homo- and Hetero- types, are the elementary unit of diode, transistor, solar cell, light-emitting-device and photodetector, that makes up the modern electronic and optoelectronic applications^1–3^. Generally, strategy in fabricating junction elements is well-defined, like through intentional chemical-doping and compositional modulation during either in-situ or back-end processes^4^. It shows fine compatibility with the state-of-art lithography and etching techniques, enabling devices shrink continuously. Lately, however, when it gets close to the physical limit (monolayers’ dimension), those junction-preparation approaches are facing with tremendous difficulties. In homogeneous PN junctions, for instance, the nature of diffusion leads to a statistical distribution of dopants in semiconductors^5^. The distribution width is comparable to or even surpass that of devices. For this reason, both academic and industry communities abandon the junction setup (complex doping strategy) in the sub-10 nm structures^5–7^, although this will dramatically increase the cost in suppressing background current/noise. The most representative cases are the junction-free transistor (FinFet^6^ and the upcoming gate-all-round^7^ architectures), in which 3D conduction channel and oxide gate have to be fabricated for a close control of current flow.

Recently, inspired by the layered structure of van der Waals (vdW) materials, interest in nanoscale carrier doping, modulation and device applications was rekindled. Particularly, three distinct routines for layered homo-junctions were carefully developed. First, electrically constructed junction^8–10^, where opposite back-gated biases or piezoelectric fields were used to accumulate electrons and holes into two adjacent areas. Second, chemically doped junction^11,12^, in which metal atoms (Cu, Co, Li and so on) are taken as p- or n-type dopants by intercalating into the vdW gap. Third, surface-transfer doped junction^13–15^, where oxidant and reductant (organic or inorganic) species are used to extract and inject electrons by an interfacial charge transfer reaction, respectively. With the success of those doping strategies, problems are also exposed. One of them is the risk of losing doping carriers in consideration of the volatile property of chemical dopants^16^ or in absence of external electric field^8–10^. Also, they are all relying on the injection of dopants or mobile-electrons, that is still in the framework of traditional manufacture process and under the law of diffusion. Therefore, the spatial resolution is far away from the monolayers’ level. In this work, we will show a striking fact that a variety of vdW layered materials (the transition metal chalcogenides and even elemental layered semiconductor, including MoS_2_, WSe_2_, MoTe_2_, black-phosphorus) don’t need external doping, they are doping themselves, gradually from p- to n- type conductance (or on opposite direction). The increasing layer-numbers of an exfoliated flake is the only critical factor that tuning this behavior. Finally, we show the great ease and freedom it offers in fabricating various devices (standard or gated diode, high V_oc_ layered solar cell and noise-signal decoupled avalanche photodetector) with remarkable performance.

## Results

In the past, great efforts have been paid in characterizing the intrinsic doping properties of vdW layered materials. The underlying technique includes gated electronics and photoluminescence. In electronic transfer curves (I_d_-V_g_), for instance, the polarity of cut-off gate-bias is the most obvious sign in identifying electron or hole conductance^8^. Optical experiment is also an option, where the luminescence peak of electron and hole charged excitons differs from each other^17^. In this study, however, we didn’t take either of them in view of two kinds of issues. First, the impact of metal-semiconductor contact, like Schottky barrier, interface states, Fermi pinning, cannot simply be excluded in electronic transport experiments^18^. Second, a number of layered materials are indirect semiconductors except in the limit of the single monolayer^19^. In this case, the luminescence efficiency would not be enough for a confident judgment.

**Fig. 1:**
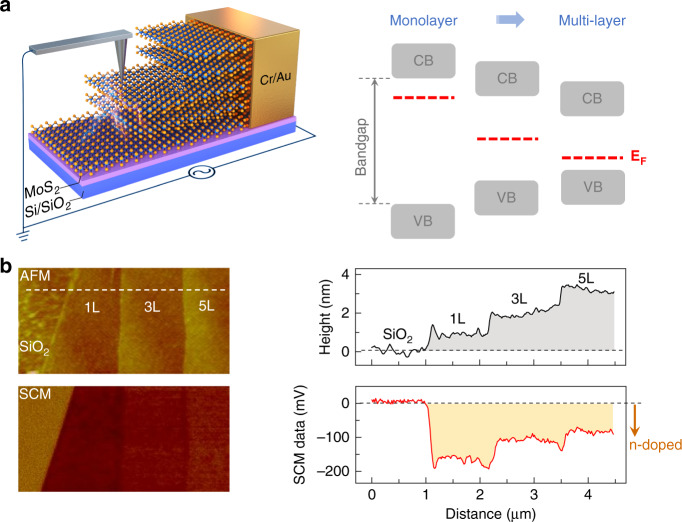
SCM experiment on vdW layered material. **a** Left panel: schematic showing the experimental setup. The sample preparation consists of mechanical exfoliation (of layered materials), electron-beam lithography and metal-contact evaporation (~60 nm Au/Cr) processes. During the measurements, the sample is powered by a 1 V 90 kHz ac bias and a conductive probe (kept virtual ground) was used to retract the localized differential capacitance signal while scanning over the device area. Right panel: evolution of the background doping characteristic of MoS_2_ with an increasing thickness. **b** AFM and SCM images of a stair-like MoS_2_ flake, including 1L, 3L and 5L segments. The profiles are derived along the white dashed line. All the SCM experiments were performed in nitrogen environment

Here, we offer a distinct routine - Scanning Capacitance Microscopy (SCM), a demonstrated technique for analyzing the polarity, concentration, and distribution of mobile charge carriers with nanoscale resolution^20^. The experimental setup is illustrated in Fig. 1a. Prior to the test, the layered material was mechanically exfoliated and transferred onto a silicon substrate (coated with ~285 nm SiO_2_). After that, standard electron-beam-lithography and Au/Cr evaporation processes were performed to ensure the electrical contact. During the experiments, a conductive probe, taken as nanoscale gate-electrode, was used to test the differential-capacitance signal (dC/dV) while scanning over the device area. Typical SCM results on the MoS_2_ flake are depicted in Fig. 1b. Both atomic-force-microscope (AFM) and optical-microscope images (Supplementary Fig. S1) identify a steplike morphology of the test flake, from mono- to triple and then fivefold layers. Correspondingly, the SCM (dC/dV) signal decreases from −160 to −100 and finally −80 mV. The negative signal denotes that few-layer MoS_2_ is intrinsically n- doped, in accordance with the previous cognition^21^. It is worth noting that zero response at the oxide layer means that it is insulating. Beyond that, an interesting phenomenon is observed: the electron concentration of MoS_2_ is stepwise decreasing with the increasing number of layers. The ever-weakening SCM signal is a solid proof.

**Fig. 2:**
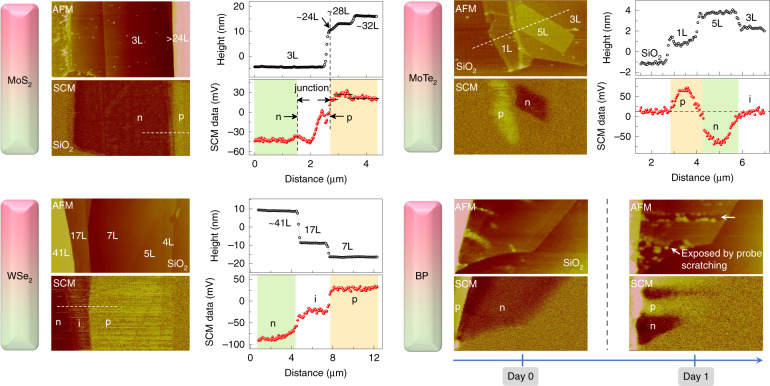
Charge-carrier distribution in layered materials. AFM and SCM images of layered MoS_2_, MoTe_2_, WSe_2_ and BP, showing the morphology, the polarity and distribution of charge carriers beneath. Note that zero response (SCM data) at oxide layer means that it is insulating, a positive/negative signal would imply a dominant response from hole/electron carriers. For BP materials, images show clear difference between the freshly exfoliated (Day 0, leading electron/hole response from few-/multi-layer BP) and oxidized states (Day 1, dominated hole response from all regions). At Day 1, we teared off the upper surface by probe scratching. The electron response was recovered in this case

Finding here deviates from the past cognition, where the layer-thickness variation should only tune the bandgap but not doping characteristic of vdW materials^19^. (A number of groups has studied the charge carrier transport of vdW heterojunctions that is featured by a varying thickness between two joint areas^22–24^) To verify this unique phenomenon and see how far it evolves (with the increasing layer thickness), we further prepared multi-layered MoS_2_ flakes. As exhibited in the upper left panel of Fig. 2, the 3L (layer) segment shows typical n-doped behavior. By contrast, both 24L, 28L, and 32L regions exhibit positive dC/dV responses, indicating an obvious p-doped characteristic. Those features imply that MoS_2_ transits from n- to p-type semiconductor with an increasing thickness. This transformation is unexpected but indeed happening, since a lateral PN junction has been formed between 3L and 24L MoS_2_. In the latter sections, we will make a detailed discussion on the charge-carrier distribution (Supplementary Fig. S6), rectifying and photoresponse properties of pristine MoS_2_ homojunction.

Obviously, the MoS_2_ flake is tuning itself from electron to hole conductance with an increasing layer-thickness. More results on MoS_2_ PN junctions, including different batches of samples, could be found in Supplementary Fig. S2. Considering that MoS_2_ is an ordinary member of semiconducting transition-metal-dichalcogenides (TMD), which shares a generalized formula (MX_2_, M is transition metal, X is chalcogen such as S, Se, Te), similar vdW layered structure and crystal phase^25^, the rule observed here might apply to other TMD materials.

Based on the assumption, we further performed experiments on layered selenide and telluride (WSe_2_ and MoTe_2_). As shown in Fig. 2, both WSe_2_ and MoTe_2_ show a polarity reversal of carrier doping with an increasing layer thickness. But note that it is in an opposite direction as compared with MoS_2_. Specifically, few layers segment is intrinsically p-type doped (consistent with the reported data, based on the transfer characteristics curves^26,27^), while multilayers become n-doped. Here we highlight the background doping behavior of MoTe_2_. 1L is p-doped and 5L has turned to n-doped. Such architecture naturally forms PN homojunction within ~ 3.5 nm’s scale (5L-thickness). More importantly, it possesses an atomically sharp boundary, that is out of the range of current techniques (As is well documented, conventional PN junction requires foreign dopants to activate neighboring electron and hole conductance, in which the PN boundary is blurred by an uncontrollable dopant interdiffusion process^5^).

Having validated that self-doped behavior is a common phenomenon in TMD, we extend the research to other two-dimensional materials. Herein, we focus on the black-phosphorus (BP), a representative of elemental layered semiconductor that has attracted great attention in the area of electronic computing, photovoltaics, and biomedicine^28^. As shown in the right bottom panel of Fig. 2, BP follows the same laws by tuning itself from electron to hole conductance (with an increasing layer-thickness). However, this status would not stay long probably due to the surface-oxidation process^29^. For details, one more night storage even in a glove box would turn few-layer BP into “p-doped semiconductor”. A feasible routine for the recovery of electron response was also researched, in which AFM probe scratches the BP surface, leaving the inner layer exposed.

For a full view of the background doping property, we make a summary on the SCM results of MoS_2_ and WSe_2_. (For more accurate instructions, those layers sandwiched in a PN homojunction are not taken into account, such as the 17L data shown in the left bottom panel of Fig. 2, since the lateral depletion or injection of charge-carriers would mislead the judgment.) For MoS_2_, 1L ≤ T(thickness) ≤ 19L leads to an obviously n-doped behavior, while the confidence interval for the p-doped property is T ≥ 24L (Supplementary Fig. S3). As a contrast, WSe_2_ retains the p-doped characteristic until 26L. And an obvious electron doping starts at T = 37L.

It was once thought that the background doping of layered materials originates from the substrate-gating effect^30^, where the buried fix-charges or mobile carriers somewhat tune the conductance of two-dimensional layers. However, this effect cannot explain the results observed here, especially when trying to interpret the opposite transition processes between MoS_2_ and WSe_2_ (MoS_2_ transits from n- to p-type semiconductors while WSe_2_ transits from p- to n-type semiconductors with an increasing layer-thickness). Lately, the role of defects/impurities on background doping is emphasized. For example, intrinsic sulfur and molybdenum vacancies are frequently found in layered MoS_2_^31,32^. They could serve as donors and acceptors, respectively^33,34^. Also, several groups argue that the commonly found Re and H impurities act as shadow donors of MoS_2_, rather than sulfur vacancies^35,36^. Although it remains controversial, such theory is helpful to understand the new finding results. The thinning process continuously tunes the bandgap and Fermi-level of vdW material, which might selectively activate the donor or acceptor levels (or modulate the contribution weight of them).

**Fig. 3:**
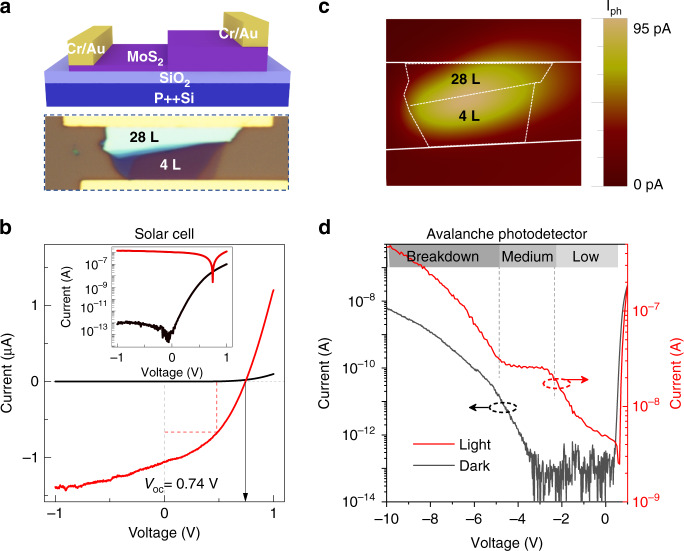
Layer PN junction and diode device based on it. **a** Schematic and optical-microscope images of a layer PN junction on MoS_2_ flake. In such device, it simply requires a MoS_2_ flake to consists of two-stepwise layer thickness, 4L and 28L. Symmetrical electrodes (Cr/Au) were deposited on both sides afterwards. **b** Dark and photo-excited IV curves of the MoS_2_ layer junction. The measurements were performed at room temperature and under an illumination of 1 µW/µm^2^ @ 520 nm. Inset: IV curves in semilog-coordinate. **c** Scanning-photocurrent-microscopy (SPCM) image of such device. The laser-beam is 520 nm in wavelength and ~1 µm in diameter. The white dashed lines mark the boundary of metal-electrodes, 4L and 28L MoS_2_. **d** Low temperature (~100 K) dark and photo-excited IV curves (under an illumination of 0.42 mW/mm^2^ @ 520 nm)

The layer-thickness-dependent doping characteristic offers great freedom and feasibility to fabricate diverse electronic and optoelectronic devices. As shown in Fig. 3a, a MoS_2_ flake consisting of two stepwise layers (4L and 28L) is a natural PN homojunction. With symmetrical electrodes deposited (Cr/Au), it can simply be fabricated into a diode device. Inset of Fig. 3b shows IV curve of such device. The cut-off current and rectification ratio are determined as ~1 pA and >10^5^, respectively. It is remarkably better than traditional vdW junctions (including chemically doped homojunction and stacked heterojunction) and comparable to the commercial silicon diode (See Supplementary Fig. S4. Following industry standards, the gate bias is absent, drain/source voltage is limited to ±1 V). To confirm that the remarkable performance comes from the homogeneous PN junction, we further performed photocurrent mapping experiments. As shown in Fig. 3c, there is no Schottky response from the Au/MoS_2_ interface. Instead, the photoresponse hot-spot centers around the 4L/28L dividing line. It means that the charge carrier separation totally relies on the PN homojunction.

Layer PN junction shows immense potential in optoelectronic applications. Because there is no chemical doping process, the junction spares itself from energic particles injection and substitutional dopant diffusion. The lattice damage and associated efficiency loss are then minimized. As depicted in Fig. 3b, when serving as a solar cell, an open circuit voltage of 0.74 V is derived, under 520 nm laser illumination @ 1 µW/µm^2^. That is a record high value achieved in the two-dimensional material (without any gate voltage), to our best knowledge (Supplementary Table 1). The power conversion efficiency is determined as 0.35%, comparable to the best value reported in 2D structures^9,18^. Beyond that, MoS_2_ layer-junction shows the potentiality of being an avalanche photodetector. As shown in Fig. 3d, the diode device can be negatively biased at three distinct states. Under a low negative bias (≤ −2.4 V), the photocurrent (under an illumination of 0.42 mW/mm^2^ @ ~100 K) slowly increases from 4.2 to 21.4 nA. It arises from the broadening of the depletion region, which thus collects more photo-carriers. For the medium voltage condition (−2.4 V to −4.5 V), the depletion region width has reached its maximum value, the photocurrent is then stabilized at ~26.1 nA. Note that the electric field is obviously enhanced at this specific region. Finally, when it reaches the threshold value (−4.5 V), the avalanche breakdown is activated. We calculated the multiplication factor of such layer-junction device, according to the equation ${{{\mathrm{M}}}} = \frac{{I_{ph} - I_d}}{{I_{bg}}}$, where *I*_*ph*_ represents the photocurrent, *I*_*d*_ is the dark current and *I*_*bg*_ denotes the net-photocurrent when M = 1. It is determined as 1.2 × 10^3^ @ −20 V (Supplementary Fig. S5), no less than that of bulk counterpart^37^.

What interests us most is that the noise and signal are decoupled in the layer-junction avalanche photodetector. Normally, the dark current should catch up with the photocurrent after break-down, making themselves little difference from each other^37^. However, in the new concept device, the dark current always falls behind the photocurrent at least one order of magnitude (Fig. 3d and Supplementary Fig. S5). It allows the device to achieve both high gain and signal-to-noise ratio. We attribute such behavior to the surface-gating effect^38^, especially considering that few-layer MoS_2_ is the voltage-drop/avalanche area and a high density of surface charges exist there (see Supplementary Fig. S6). For details, the surface charges serve as a negatively biased gate electrode, that depletes the active region and leads to a low avalanche performance in dark condition. Contrast under, the photoexcited carriers would neutralize the surface charges. It helps the device to release the full avalanche gain.

**Fig. 4:**
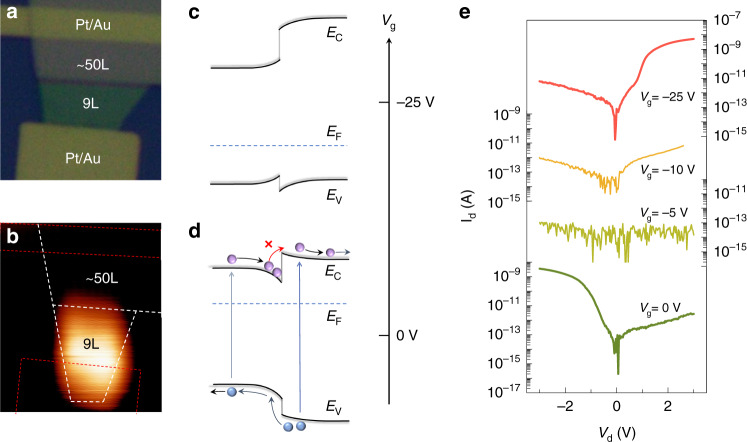
Isotype layer junction. **a** Optical microscope image of the device, showing two stairs n-doped WSe_2_, 9L and ~50L, that are electrically connected by 15 nm Pt/50 nm Au. **b** SPCM image of the device. **c**, **d** Evolution of the energy-band structure with an increasing gate bias. **e** Dependence of output curve (I_d_-V_d_) on the gate bias

At this section, we want to broaden the layer junction concept by showing a very different family member. Significantly, the layer-thickness variation would tune the bandgap of vdW material, which can be utilized to develop novel electronic devices. Figure 4a shows the optical microscopy image of such architecture. The device consists of two-stairs WSe_2_ layers, 9L and ~50L, with symmetric electrodes deposited on both sides. Have to note that the samples characterized above are all unintentionally doped. But, herein, the WSe_2_ flake is n-doped. More precisely, it was mechanically exfoliated from an intentionally n-doped WSe_2_ bulk crystal (Re dopant). SCM experiments demonstrate that both 9L and 50L WSe_2_ are n-doped in this case (Supplementary Fig. S7). The band structure is then identical to an ‘isotype n-n heterojunction’ (Fig. 4d). The alignment of Fermi levels is referring to the surface potential results, see Supplementary Fig. S8. Figure 4b shows the SPCM image of such device. One can find that multi-layer WSe_2_ is blind but few-layer responses obviously to the visible illumination. It arises from the distinct band-energy structure (Fig. 4d), where the large-conduction-band offset denies photoexcited electrons transport from multilayer.

Although isotype heterojunction was suggested since 1960s^39^, it hasn’t been widely accepted in practical applications. The band-offset (serving as electron or hole barrier) shows no superiority than that of the PN depletion region in blocking current flows. Instead, it has to treat with the lattice-mismatch related issues, including interface states, defects, stain^39,40^. Here we show that the vdW material could solve such problem and more importantly grant it an intriguing functionality: gated rectification. As shown in Fig. 4, ‘isotype heterojunction’ can be fabricated in a parent vdW material by simply changing the layer numbers of two joint areas. As there are no interface problems (between few and multi-layers), the rectification ratio is up to 10^3^ (Fig. 4e), orders higher than the bulk counterpart (n-n Ge-Si heterojunction)^40^. A more encouraging sign is that such device can be tuned from negative-conducting to forward-conducting with an increasing negative gate-bias, while the on/off currents almost keep constant in both states (Dependence of IV curves on positive gate bias is shown in Fig. S9).

Figure 4c, d illustrates the underlying mechanism. As a standard n-n junction (Fig. 4d), the Fermi-level locates at near the conduction band minimum. It thus gives rise to original negative-conducting behavior. When we give a negative gate bias (−5 V), by contrast, the layered WSe_2_ is depleted, the Fermi-level shifts close to the mid-bandgap. It then blocks the current flows in either direction (<0.1 pA). A further increase of gate bias would electrically dope WSe_2_ into hole conductance. Under such situation (typical p-p junction, Fig. 4c), the band-bending is reversed as compared with the original state. The positive-conducting mode is thus established.

Such functionality may offer an opportunity to slim the ever-growing-scale electronic circuit. Supplementary Fig. S10 shows a promising avenue, where a AND gate can be turned into a OR gate by simply applying a common gate bias (rolling over the two gated diodes). It means that the logic elements might be reconfigured dynamically, for example, to function variably in different periods, depending on the requirements.

## Discussion

In this study, we utilized SCM technique to spatially resolve the carrier distribution of two-dimensional layered materials. By this effort, a striking fact has been verified, where MoS_2_, WSe_2_, MoTe_2_, and BP are doping themselves from n- (p-) to p- (n-) type conductance with an increasing layer-thickness. Considering that the tested materials span from elemental-layered-semiconductor to transition-metal sulfide, selenide, and telluride, and there is no particular selection among them, the rules observed here might apply to other layered materials, and even the whole society.

Findings disclosed here might help to redefine the semiconductor device architecture. In the traditional manufacture procedure, there is no concept of nanoscale carrier doping since there is an inevitable diffusion process that confuses the boundary of chemical dopants. It could result in structural distortion and even failure. Here, in layered materials, each monolayer step will change the intrinsic carrier concentration, thus plays as an atomically sharp boundary. It allows devices scale down to sub-5nm’s dimension (for example, the 3.5 nm PN homojunction in MoTe_2_), which is out of the scope of the existing techniques. Equally important, the layered material will extremely simplify the manufacturing process. For instance, conventional devices have to shape both geometrical morphology and the charge-carrier profile for functionality realization. For layered material, however, we should only need to design and shape the geometrical morphology, leaving the material to depict the carrier profile on its own.

## Materials and methods

### Device fabrication

The materials of MoS_2_, MoTe_2_, WSe_2_, and BP flakes were exfoliated from the bulk crystals purchasing from *2D Semiconductors*. The MoS_2_/BP heterojunctions were fabricated by a dry transfer method inside a glovebox (N_2_ atmosphere). The Cr/Au (15/45 nm) electrode patterns were defined by standard electron-beam-lithography (performed in a FEI F50 scanning electron microscope equipped with a nanopattern generation system), thermal-evaporation and lift-off processes.

### Device characterization

The morphology of layered materials was investigated by an optical microscope (BX51, OLYMPUS). The electrical transport measurements were carried out by a semiconductor parameter analyzer (B1500, Agilent) in a probe station (Lake Shore TTPX). A flow of liquid nitrogen was provided to cool the device to the target temperature. For the scanning-photocurrent-microscopy (SPCM) measurements, a focused laser spot (520 nm in wavelength, ∼1 μm in diameter) was controlled to scan over the device area. With the output photocurrent recorded in real-time, the SPCM images thus show the contribution of each segment to the overall photoresponse.

### Setup of SCM experiments

Scanning capacitance microscopy, equipped with a Multimode-Nanoscope-*IV* controller, was utilized to characterize the charge-carrier distribution. Probes coated with conductive diamond (force constant: 21–98 N/m, microscopic tip radius: ~10 nm) was chosen for such contact-mode measurements. It takes as a nanoscale gate-electrode, which retracts the differential capacitance signal while scanning over the device area. During the experiments, the probe was kept virtual ground, while an AC bias voltage of 1.0 V at 90 kHz was applied to the device electrode.

## Supplementary information

Supplementary information
