# Supplementary material for: Pristine PN junction toward atomic layer devices
Source: Light Sci Appl. 2022 Jun 6;11:170. doi: 10.1038/s41377-022-00814-8 (PMC9167816; doi:10.1038/s41377-022-00814-8)
Supplement: Supplementary file 1 — Supplementary information [file 41377_2022_814_MOESM1_ESM.docx]

Supplementary Materials for

Pristine PN junction toward atomic layer devices

*Hui Xia^1,2^*^†^*, Man Luo^3^*^†^*, Wenjing Wang^1,2^, Hailu Wang^1,2^, Tianxin Li^1,2*^, Zhen Wang^1,2^, Hangyu Xu^1,2^, Yue Chen^1,2^, Zhou Yong^1,2^, Fang Wang^1,2^, Runzhang Xie^1,2^, Peng Wang^1,2^, Weida Hu^1,2*^, Wei Lu^1,2,4*^*

^1^State Key Laboratory of Infrared Physics, Shanghai Institute of Technical Physics, Chinese Academy of Sciences, Shanghai 200083, China.

^2^University of Chinese Academy of Sciences, Beijing 100049, China.

^3^Jiangsu Key Laboratory of ASIC Design, School of Information Science and Technology, Nantong University, Nantong, Jiangsu 226019, China.

^4^School of Physical Science and Technology, ShanghaiTech University, Shanghai 201210, China.

*Corresponding author:

[txli@mail.sitp.ac.cn](mailto:txli@mail.sitp.ac.cn) (T. Li); [wdhu@mail.sitp.ac.cn](mailto:wdhu@mail.sitp.ac.cn) (W. Hu); [luwei@mail.sitp.ac.cn](mailto:luwei@mail.sitp.ac.cn). (W. Lu)

†H. Xia, M. Luo contributed equally to this work.

**This Word file includes:**

Supplementary Text

Figs. S1 to S10

Tables S1 and S2

References (1 to 12)

**Supplementary Text**

**Simulated dC/dV profiles of MoS_2_ homojunction.**

At this section, we perform a quantitative interpretation on the carrier distribution (dC/dV profiles) of a lateral MoS_2_ homojunction (shown in the upper left panel of Fig.2). To that end, a numerical model is established with a commercial software package (SENTAURUS-TCAD). Technically, the conductive probe is treated as a mobile 6nm-scale gate electrode; the carriers and electric field distributions are simulated by coupling numerical solutions of Poisson equation and electron hole continuity equation; dC/dV response of the layered material is calculated by applying the small-signal ac analysis model^1^. Some key parameters, including bandgap, electron affinity, mobility of 3L and 24L MoS_2_, are summarized in Supplementary Table 2. First, we assume an ideal situation where no surface effect exists. The junction region then shows a flat zero dC/dV response. Note that such characteristics is frequently reported in bulk Si PN junction^2^, denoting a low carrier concentration in the depletion region. However, it deviates from the reality of MoS_2_. Later, we modified the model by adding surface charges model. The simulation then well reproduces the "double dip" dC/dV profile (Extended Data Fig.6). This contrast verifies the existence of surface charges/states in layered MoS_2_.

Simulations help to retract the fingerprint information of MoS_2_ homojunction. The surface charge density is as high as -1.0×10^10^ and + 2.1×10^11^cm^-2^ in n- and p-doped regions, respectively. It results in the distinct carrier distribution. Specifically, feature located at -1.2 µm (Extended Data Fig.6) arises from a rapid decline of negative surface charges, the electron concentration thus recovers to a high level. Feature at -0.25 µm comes from the positive surface charges, which helps to accumulate excess electrons. Moreover, the electron concentration of few-layer MoS_2_ is determined as 1.9×10^10^ cm^-2^, close to the previous reports^3^, 3.61×10^10^ cm^-2^, derived from the transfer IV curves). The hole concentration of 24L MoS_2_ is derived as 3.4×10^12^ cm^-2^.


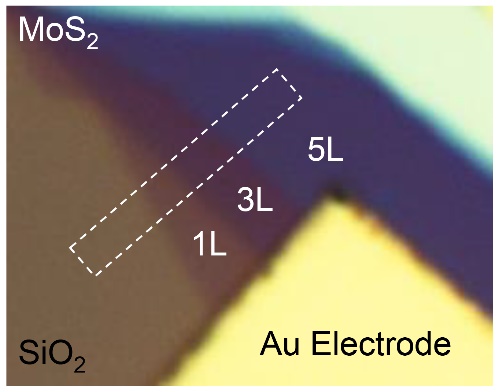


**Fig.S1. Optical-microscope-image of the MoS_2_ flake (characterized in Fig.1b).** The white dotted outline indicates the test area, containing SiO_2_ substrate, mono- to triple and then fivefold layers MoS_2_. Note that the Au film serves as a common electrode as it electrically connects to 1L, 3L and 5L MoS_2_ simultaneously.


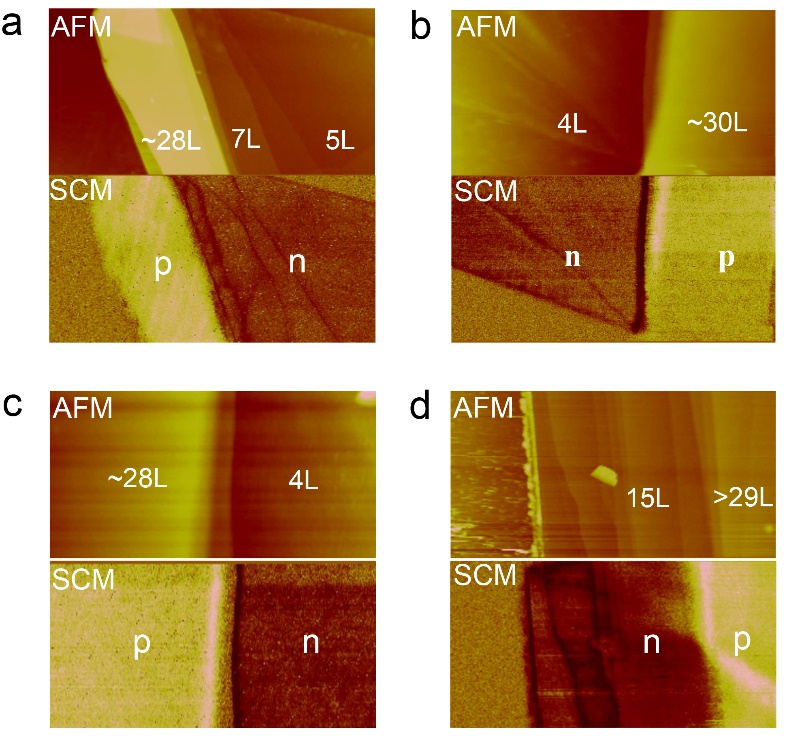


**Fig.S2. More results on MoS_2_ layer-junctions, including different sources of samples.**


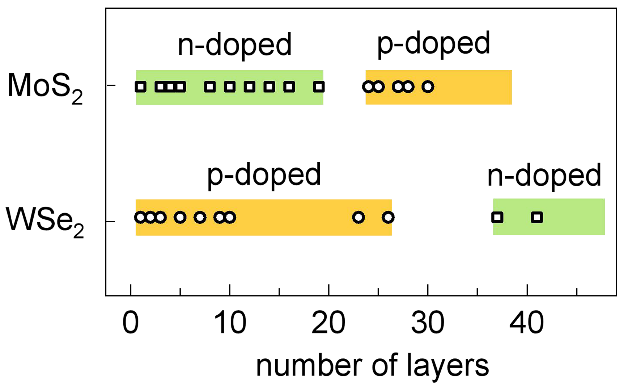


**Fig.S3. Confidence interval thickness for n- and p- doped MoS_2_ and WSe_2_**. Each scatter sign represents an authentic data observed in SCM experiments.

**Fig.S4. Comparison of rectification ratio and cut-off current of 2D heterostructures MoS_2_/AsP^4^, WSe_2_/Bi_2_Te_3_^5^, n-InGaAs/BP^6^, WSe_2_/ReS_2_^7^, MoS_2_/GaAs^8^, ReS_2_/ReSe_2_^9^, MoTe_2_/SnSe_2_^10^, homojunction MoSe_2_^11^ and conventional bulk materials Si^12^.**


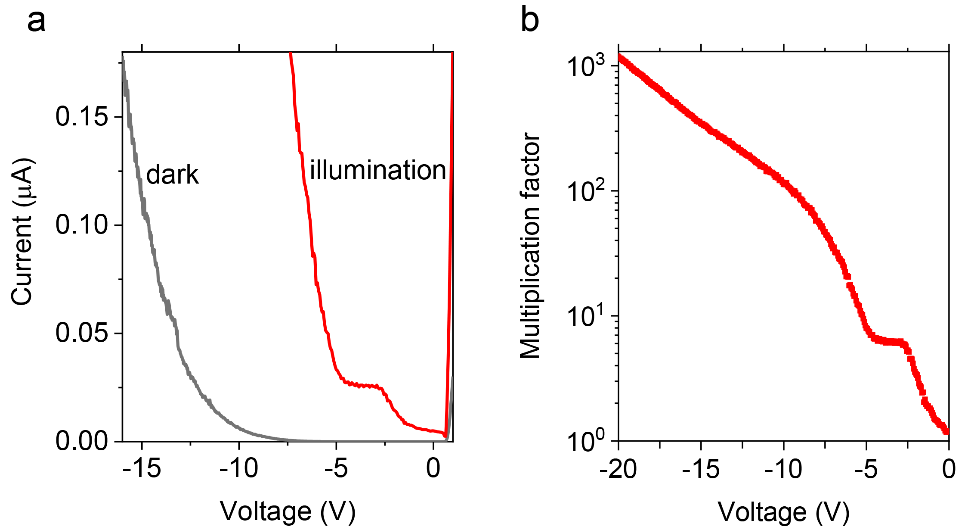


**Fig.S5. Avalanching performance of MoS_2_ layer-junction. a**, Dark and photoexcited IV curves of MoS_2_ layer-junction (shown in Fig.3c) in linear coordinate. The measurements were performed at ~100K and under an illumination of 0.42 mW/mm^2^ @520nm. **b**, Dependence of multiplication factor on the applied negative bias. The white dotted outline indicates the test area, containing SiO_2_ substrate, mono- to triple and then fivefold layers MoS_2_. Note that the Au film serves as a common electrode as it electrically connects to 1L, 3L and 5L MoS_2_ simultaneously.


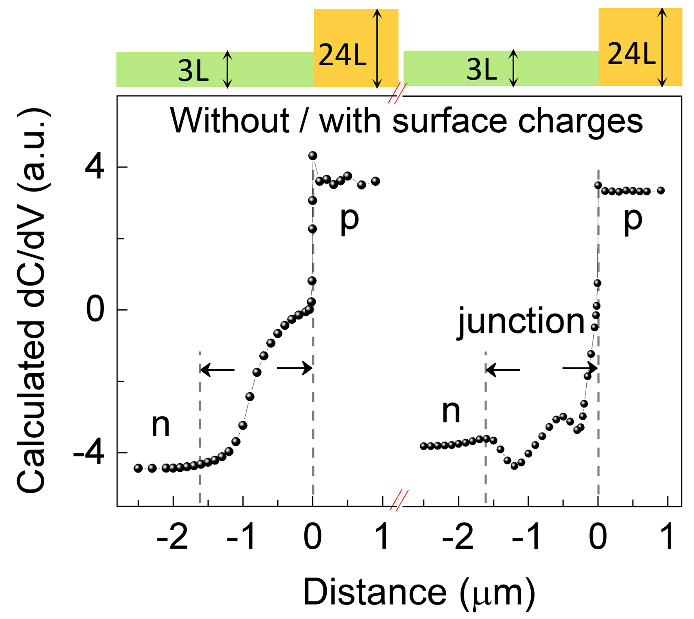


**Fig.S6. Simulated dC/dV profiles of MoS_2_ homojunction.** 3L/24L MoS_2_ is set n-/p- doped, referring to the experiments (Fig.2). Parameters adopted are listed in Supplementary Table 2.


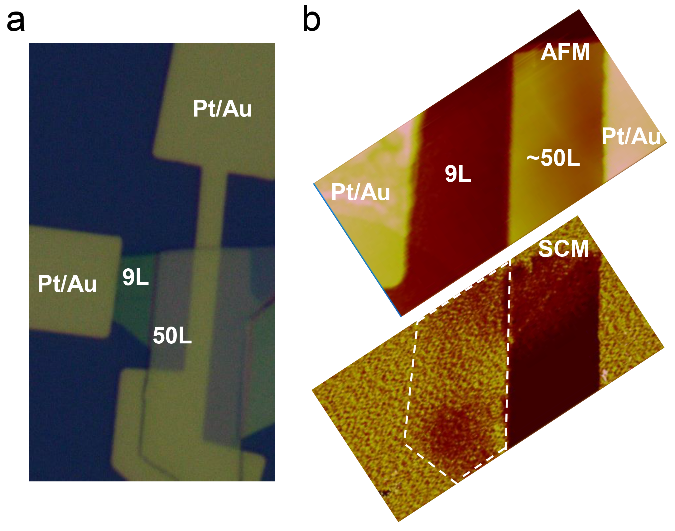


**Fig.S7. SCM experiments on isotype WSe_2_ layer junction. a**, Optical-microscope image of the device. **b**, Corresponding AFM and SCM results. Both 9L and 50L WSe_2_ layers exhibit negative dC/dV response, indicating a dominant electron conductance.


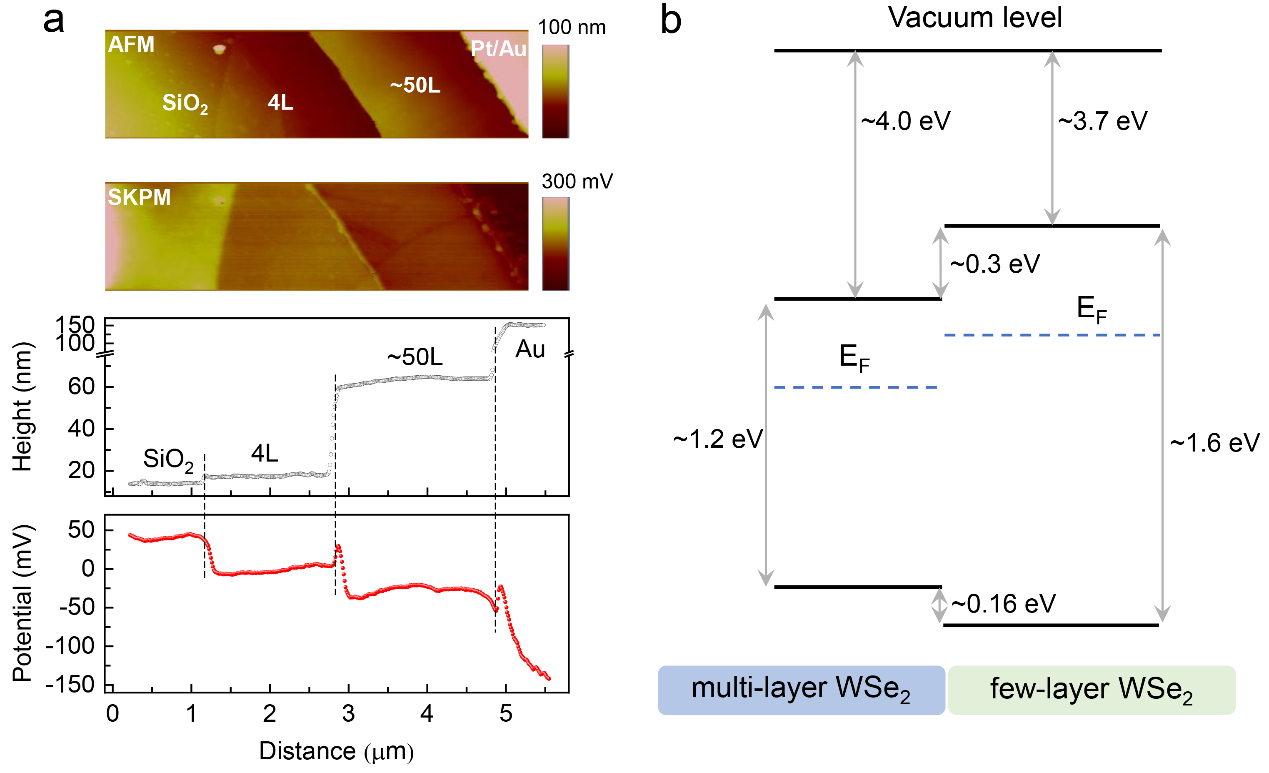


**Fig.S8. Band structure of the isotype WSe_2_ layer junction. a**, SKPM result of the device. Obviously, the surface-potential/Fermi-level of 50L WSe_2_ is lower than that of 4L counterpart. **b**, Schematic showing the band structure of the junction before contact.


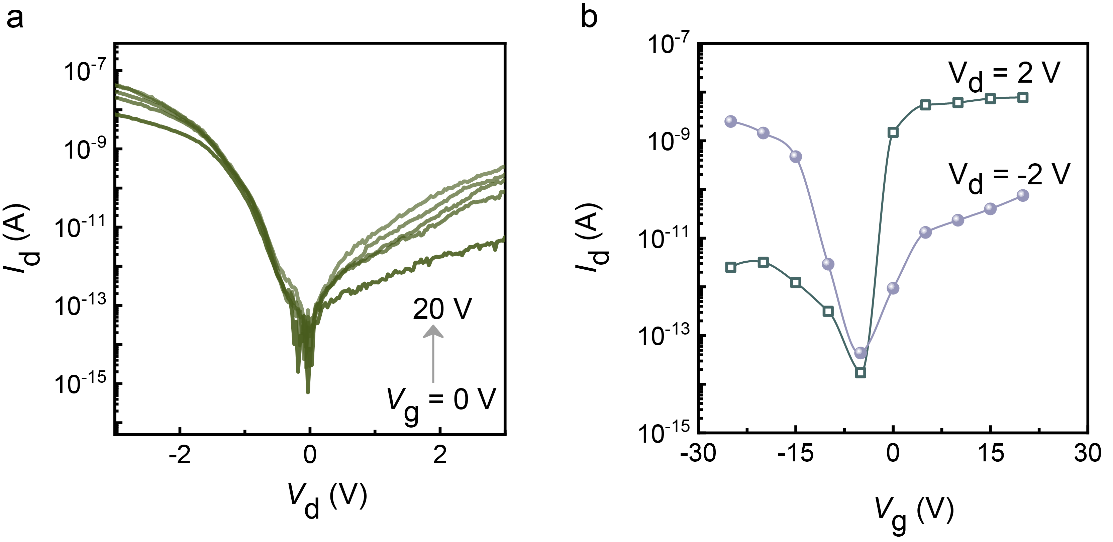


**Fig.S9. Electrical characterization of isotype WSe_2_ layer junction (shown in Fig.4). a**, Output curves under a varying positive-gate-bias. **b**, transfer curves derived from **a** and Fig.4e.

- Effect of drain bias on the photocarriers transport.

Under forward bias, the electron barrier is lowered. Photocarriers generated at both sides contribute to the device photoresponse. Thus, the photocurrent hot spot would cover the whole van der Waal region.

Under negative bias, the electron barrier is significantly arised. Photocarriers generated at multi-layer are much more difficult to be collected. Thus, the photocurrent hot spot should localize at the few-layer WeSe_2_.

- Dependence of gate bias on the photocarriers transport.


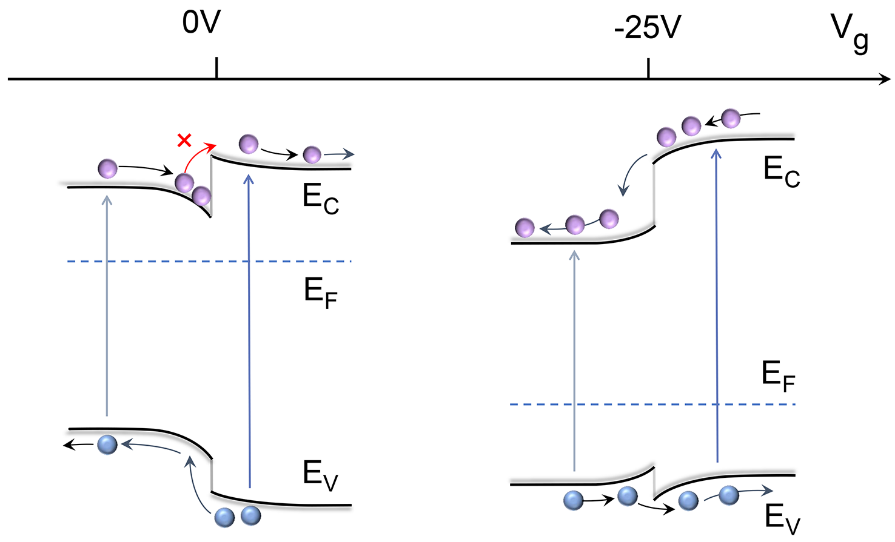


Figure S10. Effect of gate bias on the photocarriers transport.

When we give a negative gate bias (-25V), few-layer and multi-layer WSe_2_ are both electrically doped to hole conductance (typical p-p junction, Figure R3). The band-bending is then reversed as compared with the original state. Under such situation, photocarriers generated at both layers can be collected efficiently; the photocurrent hot spot would cover the whole van der Waal region.

**Fig.S11. Potential application in logic circuit**. where the diode can be electrically reversed, allowing a AND gate to turn into a OR gate.

**Supplementary Table 1. Photovoltaic effect of vdW junction**

| Device structure | *V*_oc_（V） | | Reference |  |
| --- | --- | --- | --- | --- |
| Layer-junction | MoS_2_ layer-junction | | 0.74 | This work |
| Chemically doped homojunction | chemically doped vertical MoS_2_ p–n homojunction | | 0.6 | Ref.13 |
|  | lateral WS_2_ p−n homojunction doped and passivated by M_o_O_x_ | | 0.681 | Ref.14 |
| Vertically stacked heterojunction | MoS_2_/AsP vertical heterojunction | | 0.61 | Ref.4 |
|  | WSe_2_/Bi_2_Te_3_ vertical p−n heterojunction | 0.25 | | Ref.5 |
|  | MoS_2_/WSe_2_ vertical p−n heterojunction | 0.5 | | Ref.15 |

In this work, the photovoltaic efficiency of layer-junction is derived as 0.35%, comparable to the best value reported in layered structures (Nat. Nanotech. 2014, 9, 257-261; Nature 2018, 557, 696-700).

Note that our device was examined at zero gate bias, while a number of devices were operated on a high gate voltage (see Supplementary Table 2).


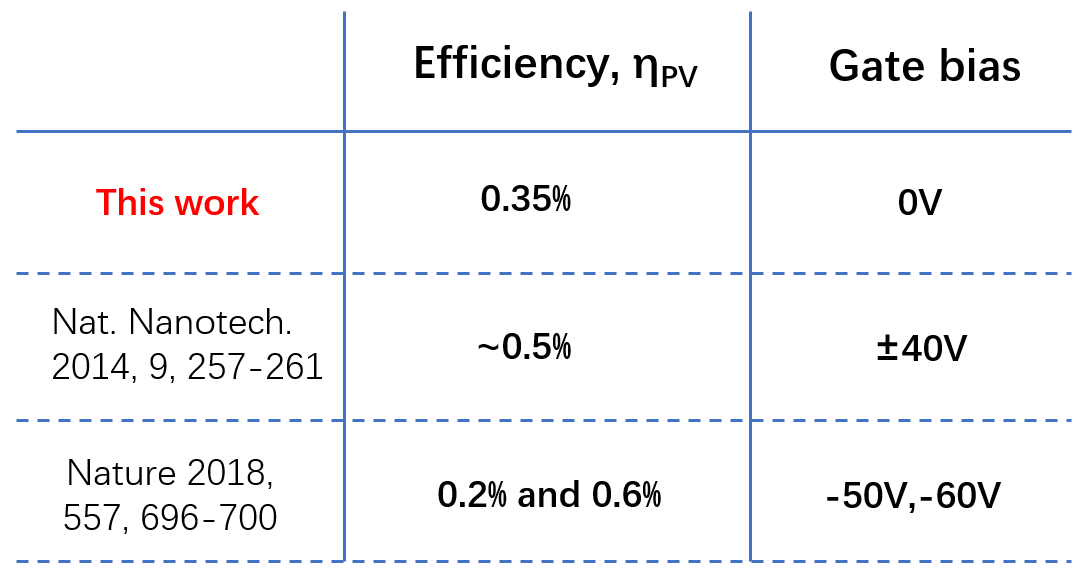


Supplementary Table 2. Comparison of the photovoltaic efficiency in different works.


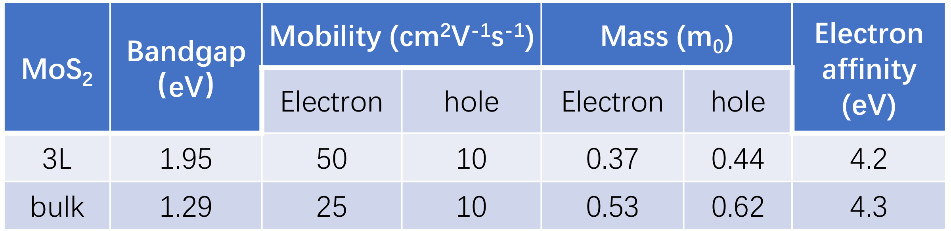


**Supplementary Table 3. Some key parameters used in numerical simulation, including bandgap, electron affinity, mobility of 3L and 24L MoS_2_**

**Supplementary Reference:**

1 Xia, H. *et al.* Nanoscale imaging of the photoresponse in PN junctions of InGaAs infrared detector. *Sci. Rep.* **6**, 1-8, (2016).

2 Jiang, C. S., Heath, J. T., Moutinho, H. R. & Al-Jassim, M. M. Scanning capacitance spectroscopy on n(+) -p asymmetrical junctions in multicrystalline Si solar cells. *J. Appl. Phys.* **110**, (2011).

3 Kwak, J. Y. *et al.* Electrical characteristics of multilayer MoS_2_ FET's with MoS_2_/graphene heterojunction contacts. *Nano Lett.* **14**, 4511-4516, (2014).

4 Wu, F. *et al.* High efficiency and fast van der Waals hetero-photodiodes with a unilateral depletion region. *Nat. Commun.* **10**, 1-8, (2019).

5 Liu, H. *et al.* Self-powered broad-band photodetectors based on vertically stacked WSe_2_/Bi_2_Te_3_ p-n heterojunctions. *ACS Nano* **13**, 13573-13580, (2019).

6 Lee, Y. *et al.* Gate-tunable and programmable n-InGaAs/black phosphorus heterojunction diodes. *ACS Appl. Mater. Interfaces* **11**, 23382-23391, (2019).

7 Varghese, A. *et al.* Near-direct bandgap WSe_2_/ReS_2_ type-II pn heterojunction for enhanced ultrafast photodetection and high-performance photovoltaics. *Nano Lett.* **20**, 1707-1717, (2020).

8 Jia, C. *et al.* A self-powered high-performance photodetector based on a MoS2/GaAs heterojunction with high polarization sensitivity. *J. Mater. Chem. C* **7**, 3817-3821, (2019).

9 Cho, A.-J., Namgung, S. D., Kim, H. & Kwon, J.-Y. Electric and photovoltaic characteristics of a multi-layer ReS_2_/ReSe_2_ heterostructure. *APL Mater.* **5**, 076101, (2017).

10 Li, C. *et al.* WSe_2_/MoS_2_ and MoTe_2_/SnSe_2_ van der Waals heterostructure transistors with different band alignment. *Nanotechnology* **28**, 415201, (2017).

11 Yang, Y. J., Huo, N. G. & Li, J. B. Gate tunable photovoltaic effect in a MoSe_2_ homojunction enabled with different thicknesses. *J. Mater. Chem. C* **5**, 7051-7056, (2017).

12 Guliants, E. A., Ji, C. H., Song, Y. J. & Anderson, W. A. A 0.5-um-thick polycrystalline silicon Schottky diode with rectification ratio of 10^6^. *Appl. Phys. Lett.* **80**, 1474-1476, (2002).

13 Li, H.-M. *et al.* Ultimate thin vertical p-n junction composed of two-dimensional layered molybdenum disulfide. *Nat. Commun.* **6**, 1-9, (2015).

14 Nazif, K. N. *et al.* High-Performance p-n Junction Transition Metal Dichalcogenide Photovoltaic Cells Enabled by MoOx Doping and Passivation. *Nano Lett.* **21**, 3443-3450, (2021).

15 Lee, C.-H. *et al.* Atomically thin p-n junctions with van der Waals heterointerfaces. *Nat. Nanotech.* **9**, 676-681, (2014).
